# Role of Microglial Cells in the Pathophysiology of MS: Synergistic or Antagonistic?

**DOI:** 10.3390/ijms24031861

**Published:** 2023-01-17

**Authors:** Hubert Mado, Monika Adamczyk-Sowa, Paweł Sowa

**Affiliations:** 1 Department of Neurology, Faculty of Medical Sciences in Zabrze, Medical University of Silesia in Katowice, 40-055 Katowice, Poland; 2 Department of Otorhinolaryngology and Oncological Laryngology, Faculty of Medical Sciences in Zabrze, Medical University of Silesia in Katowice, 40-055 Katowice, Poland

**Keywords:** multiple sclerosis, cytokines, microglia, neuroimmunology, disease-modifying therapies

## Abstract

Many studies indicate an important role of microglia and their cytokines in the pathophysiology of multiple sclerosis (MS). Microglia are the macrophages of the central nervous system (CNS). They have many functions, such as being “controllers” of the CNS homeostasis in pathological and healthy conditions, playing a key role in the active immune defense of the CNS. Macroglia exhibit a dual role, depending on the phenotype they adopt. First, they can exhibit neurotoxic effects, which are harmful in the case of MS. However, they also show neuroprotective and regenerative effects in this disease. Many of the effects of microglia are mediated through the cytokines they secrete, which have either positive or negative properties. Neurotoxic and pro-inflammatory effects can be mediated by microglia via lipopolysaccharide and gamma interferon. On the other hand, the mediators of anti-inflammatory and protective effects secreted by microglia can be, for example, interleukin-4 and -13. Further investigation into the role of microglia in MS pathophysiology may perhaps lead to the discovery of new therapies for MS, as recent research in this area has been very promising.

## 1. Introduction

Multiple sclerosis (MS) is a chronic disease of the central nervous system (CNS) with a presumed autoimmune basis [1,2,3,4,5,6,7]. It is an inflammatory disorder characterized by progressive neurodegeneration [1,2,3,4,5]. It is estimated that there are currently about 2.8 million people with the disease worldwide (35.9 patients per 100,000 individuals). Annually, 2.1 new cases of MS are diagnosed per 100,000 individuals [8]. Therefore, MS is the most common demyelinating disease [9]. There are significant geographic differences in the prevalence of this condition, with the highest incidence in North America and Europe (>100 per 100,000 population) and the lowest in sub-Saharan Africa and East Asia (2 per 100,000 population) [9]. However, it is important to be concerned with potential gaps in the availability of reliable data in areas with the lowest incidence of the disorder, especially compared to North America and Europe. Nevertheless, the incidence of the disease is increasing [8,10]. This condition is twice as common in women and the mean age at diagnosis is 32 years [8]. In some countries, including those in Europe, the incidence is three times higher in women in the case of relapsing-remitting MS (RRSM) [9]. Despite its significant prevalence, MS is an incurable condition. Therefore, studies are warranted to search for new pathophysiological aspects that may allow the search for new treatment options. Presumably, a better understanding of the role of microglia in the pathophysiology of this condition could serve this purpose. Therefore, the aim of this review is to summarize the current state of knowledge about microglia in the pathophysiology of MS.

## 2. Fundamentals of MS Pathogenesis

The underlying mechanism of the pathogenesis of MS is related to an impaired immune response. B lymphocytes, which are responsible for the production of autoantibodies and cytokines, are involved in the pathophysiology of this disease [11,12]. B cells also mediate the effects exerted by antigen-presenting cells. The result is the activation of T cells, which is thought to be a key element in the pathomechanism of this condition [11]. It results in the infiltration of the brain and spinal cord by inflammatory cells. Areas of CNS demyelination also develop [13,14,15,16]. Initially, the inflammatory process predominates. However, later the degenerative process is predominant. Neuronal, axonal and synaptic damage and glial cell destruction are reported [13,14,15,16]. Macrophages in the CNS, known as microglia, have been shown to play a significant role in these pathological processes [5,17,18,19].

## 3. What Are Microglia?

Microglia are glial cells in the brain and spinal cord [20]. Microglial cells account for about 7% of the brain cells [21]. These cells originate from the yolk sac and populate the brain mesenchyme. Microglia continuously renew themselves in the CNS, and there is no replenishment from peripheral monocyte precursors [22]. Microglia were first identified about a hundred years ago [23]. Franz Nissl, who developed the Nissl staining, and William Ford Robertson were the first researchers to describe microglial cells [24]. They did it independently during their experimental histological studies at the end of the 19th century and the beginning of the 20th century [24]. In turn, Santiago Ramón y Cajal was the first researcher to define another cell type besides neurons and astrocytes, i.e., the so-called “third element” [25]. Subsequently, Pío del Río Hortega, a student of Santiago Ramón y Cajal, was the first scientist to term these newly discovered cells microglia, which occurred around 1919 [25]. In the course of his research, Hortega concluded that the resting microglia could undergo transformation into an amoeboid form, which occurs in various types of CNS pathology [25]. Initially, it was thought that microglia cells originated from the neuroectoderm [23]. Currently, it is known that this is not true and that microglial cells are CNS macrophages [23].

Nevertheless, although the evidence for it came much later, Río Hortega also suggested a potential mesodermal origin of microglia [25] and also noted their phagocytic abilities and suggested their functional similarity to macrophages [25]. In contemporary animal studies, microglial cells were shown to be generated very early in embryogenesis, long before the formation of other glial cells [23]. In mouse studies, microglial cells were found to develop initially from c-Kit^lo^ CD41^lo^ progenitor cells [23]. These fetal yolk sac-derived macrophages populate the CNS at very early developmental stages [26]. Once they enter the CNS, they spread throughout the parenchyma and undergo many functional and morphological transformations [26]. As mentioned earlier, currently, it is assumed that microglia are a long-lived remnant of early primitive hematopoiesis in the CNS, and in the course of postnatal life, their pool is not replenished with cells of myeloid origin [27]. Microglial premacrophages derived from early erythroid marrow precursors are thought to be formed independently of the transcription factor Myb [23].

So far, several morphological forms of microglia have been recognized [28]. Under physiological conditions, a ramified form of microglia that represents the resting form of these cells is present in the healthy brain [28]. Ramified microglia are found in the CNS in the absence of dead cells or foreign material [28]. These cells are characterized by a small cell body and long thin and branched processes [26,28]. The cell body of ramified microglia remains stationary within a given territory nearly 15–30 µm wide, with a little overlap between neighboring territories [26]. In turn, with the help of their movable processes, these microglial cells continuously explore the specific territory of a given single unit [26,28]. Such movement of microglial processes occurs at a speed of about 1.5 µm/min, which makes them the fastest-moving structures within the brain [26]. As a result, the brain parenchyma can be completely explored by microglia within hours [26]. This form of microglia does not have a phagocytic function but instead recognizes immune threats, thus participating in maintaining the CNS homeostasis [28,29,30,31]. However, in response to threat or damage, ramified microglia can be transformed into an activated form at any time [26,28]. Microglial activation, which is a complex process, leads to a change in the phenotype and function of these cells [26]. In the course of such activation, microglial cells acquire the ability to present antigens and mediate inflammation, as well as phagocytic properties [28,30]. Many of these phenotypes are disease-specific [26]. As a result of such activation in the case of disease, microglia can be transformed into a neurotoxic form, which, according to Butt and Verkhtrasky, constitutes a pathological factor per se [26]. There are also microglia with amoeboid morphology [32]. This type of microglia occurs mainly during brain development and remodeling [32]. Ameboid microglia are characterized by their ability to move throughout the neural tissue, functioning as scavengers in phagocytosis [32]. However, unlike activated microglia, ameboid microglia lack the ability to present antigens and mediate inflammation [32].

## 4. Microglia Function and Phenotypes

The resident macrophages serve as the first and leading active immune defense of the CNS [33]. In addition, microglia maintain the CNS homeostasis under pathological and healthy conditions [34]. Microglia behave as “controllers” that monitor the processes in the CNS microenvironment and the normal functioning of neuronal cells, dendrites and axons [5,35]. These CNS macrophages clear the CNS of unnecessary matter, such as atherosclerotic plaques and infectious agents [36]. In addition, microglia phagocytose dead cells and their parts [5,35]. They recognize foreign bodies, phagocytose them and function as antigen-presenting cells that activate T cells [30,36].

The nature of microglia is highly plastic. Depending on the situation, microglia can exert different and often opposing effects [5]. Microglia can be divided into two phenotypes with opposite functions, i.e., the classical type (M1) and the alternative one (M2) [37,38]. They can transform from the M1 to the M2 phenotype and vice versa [37,38]. Some researchers refer to this phenomenon as M3 microglia, which switches to the M1 or M2 phenotype depending on the inducing stimuli (Malyshev). Activation of M1 microglia typically occurs via lipopolysaccharide and interferon gamma (IFN-γ) [37,39]. M1 microglia are responsible for neurotoxicity, the release of inflammatory mediators and the induction of inflammation [37]. On the other hand, M2 microglia are responsible for the release of anti-inflammatory mediators and for anti-inflammatory effects, as well as have neuroprotective properties [37]. M2 microglia can be divided into M2a, M2b and M2c [40]. M2a is a phenotype with anti-inflammatory, phagocytic and antiparasitic activities. It is also involved in collagen formation and tissue repair [40,41,42]. The activation of M2a is induced by anti-inflammatory cytokines, such as IL-4 and IL-13 [37,39,40,42]. M2b microglia are implicated in regulatory functions and are activated by the fusion of TLR and FCγ receptors [40,43,44,45]. In the polarized state, M2b microglia show similar properties to those of M1. However, they may also be responsible for the release of anti-inflammatory IL-10 and the recruitment of regulatory T cells [40,46]. M2c microglia are activated by IL-10 with TGF-β and glucocorticoids [40,41,47]. M2c microglia have an immunosuppressive function, while in their polarized state, which occurs under the influence of IL-10, they participate in immunoregulation, matrix remodeling and tissue repair [40,41,47,48]. Therefore, they have a dual role. The comparison of the roles of different microglial phenotypes is presented in Table 1.

## 5. Activation of M1 and M2 Microglia

**Figure 1:**
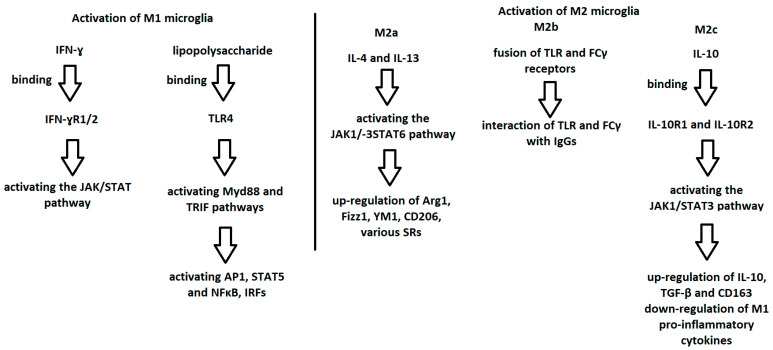
Graphic demonstration of the mechanism of microglia activation to the M1 and M2 phenotypes.

As previously mentioned, the polarization of microglia from the “resting” state toward the pro-inflammatory M1 phenotype is induced by IFN-γ or the endotoxin lipopolysaccharide of Gram-negative bacteria [40] (Figure 1). IFN-γ binds to IFN-γ receptors 1 and 2 (IFN-γR1/2), in effect activating the JAK/STAT pathway [40,49]. This in turn leads to the phosphorylation and nuclear translocation of STAT1 and other IRFs [40,49]. Lipopolysaccharide is a ligand for Toll-like receptor 4 (TLR4) [40]. As a result, TLR4 binds to its coreceptors resulting in the activation of pro-inflammatory transcription factors such as AP1, STAT5, NFκB, IRFs [40,50,51]. This process is mediated by pathways dependent on myeloid differentiation primary response protein 88 (MyD88) and TIR domain-containing adaptor inducing IFN-β (TRIF) [40,50,51].

The polarization of microglia toward the anti-inflammatory phenotype of M2a is triggered by IL-4 and IL-13 [40] (Figure 1). This involves the activation of the JAK1/3–STAT6 pathway, which leads to the up-regulation of microglia cell surface markers such as Arg1 (arginase-1), Fizz1 (found in inflammatory zone1), YM1 (chitinase-like protein), CD206 (mannose receptor) and various SRs (scavenger receptors) [40,48,52]. In the case of the M2b phenotype, activation is via a fusion of TLR and FCγ receptors [40,43,44,45]. Then, there is an interaction between these receptors and IgG immunoglobulins [40]. The polarization of microglia toward the M2c phenotype can occur as a response to the effect of IL-10 with TGF-β and glucocorticosteroids [40,41,47]. The interaction of IL-10 and the receptors 1 (IL-10R1) and 2 (IL-10R2) for IL-10 is followed by the activation of the JAK1/STAT3 pathway [40,41]. As a result, there is an up-regulation of IL-10, TGF-β and CD163 with a concomitant inhibition of the secretion of pro-inflammatory cytokines characteristic of the M1 phenotype [40,41] (Figure 1).

## 6. Microglia and MS

**Figure 2:**
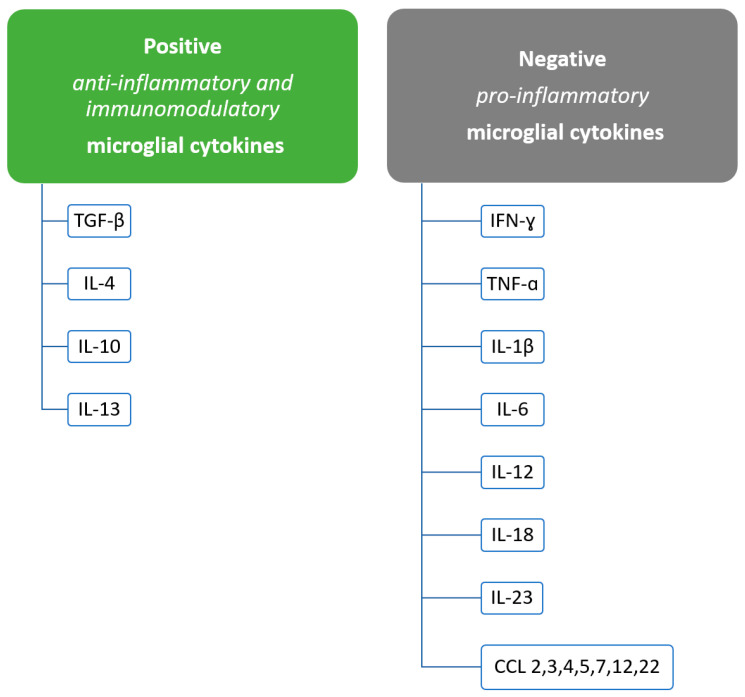
Division of microglial cytokines depending on their positive or negative effects in the pathogenesis of MS.

The dual role of microglia has been demonstrated in the pathogenesis of MS, where they exert both beneficial and negative effects [5]. During inflammation in the CNS, e.g., in the pathogenesis of MS, microglia also have secretory and modulatory functions. They can secrete pro-inflammatory cytokines, chemokines and factors that promote regeneration, such as anti-inflammatory cytokines and molecules with immunomodulatory functions [5,53,54,55]. Soluble factors secreted by microglia that participate in negative effects in the pathogenesis of MS include IFN-γ, tumor necrosis factor-alpha (TNF-α), reactive oxygen species, interleukin 1β (IL-1β), IL-6, IL-18, IL-12 and IL-23 and also chemokines such as CCL2, CCL3, CCL4, CCL5, CCL7, CCL12 and CCL22 [5,17,56,57] (Figure 2). In contrast, the molecules by which microglia mediate beneficial effects in MS include IL-4, IL-10, IL-13 and transforming growth factor beta (TGF-β) [17,58,59] (Figure 2).

Microglia are involved in pathological processes in both white matter and gray matter [5]. These processes include synaptic dysfunction and axonal and neuronal degeneration [5,60]. The aspect of microglial participation in neurodegeneration in MS should be emphasized. Demyelination, which affects the gray and white matter, can be reversed by remyelination, in which microglia also participate incidentally through secreted cytokines [5,61,62]. In turn, neurodegeneration of the gray matter is irreversible and occurs very early in MS and is largely responsible for permanent disability [5,61,62]. This aspect is all the more important because current disease-modifying therapies (DMTs) only reduce the loss of white matter but have limited properties in terms of significant reduction or prevention of gray matter neurodegeneration [5,63]. 

## 7. Beneficial Properties of Microglia in MS

**Figure 3:**
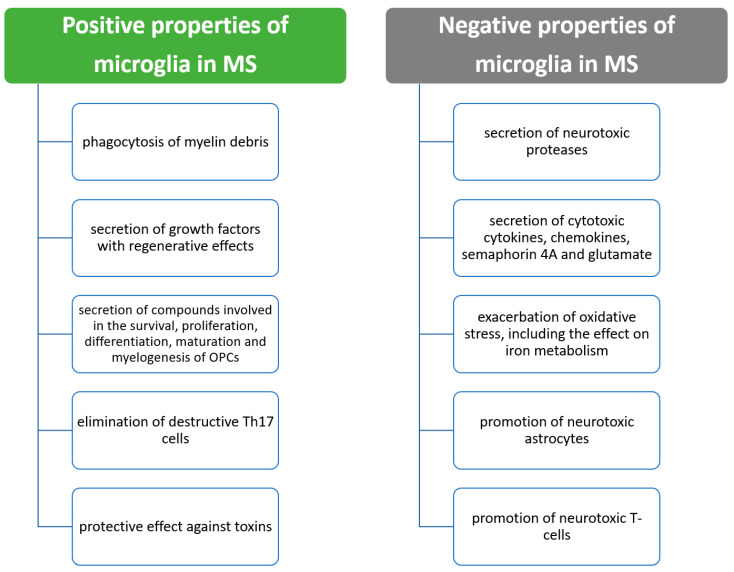
Beneficial and negative effects of microglia in MS.

Apart from the beneficial properties of microglia in terms of homeostasis before the development of MS, microglia have many profitable properties already after the formation of MS lesions [64]. It has been shown that after demyelination, microglia perform an important function in the removal by phagocytosis of myelin debris with inhibitory effects. This process is mediated by TREM-2, MerTK, CX3CR11 and CD36, among others [64,65,66,67]. In addition, microglia secrete a number of growth factors that promote recovery from the demyelination that has occurred [64]. Many positive and negative effects of microglial cytokines are exerted through anti-inflammatory and immunomodulatory cytokines such as IL-4, IL-10, IL-13 and TGF-β [17,58,59]. Among the factors secreted by microglia that have been demonstrated in animal models or tissue culture, we can further include transglutaminase, semaphorin 3F, activin-A, brain-derived neurotrophic factor and insulin-like growth factor-1 [54,64,68,69]. These factors participate in the survivability, proliferation and differentiation of oligodendrocyte precursor cells (OPCs). In addition, through neuropilin-1, microglia are involved in the maturation and myelinogenesis of OPCs [64,69]. Furthermore, in mice with autoimmune encephalomyelitis, microglia have been shown to be involved in the elimination of destructive T helper 17 cells (Th17 cells) [64,70]. Finally, in MS, microglia have a protective function against toxins that promote the pathogenic process [64,67]. The positive effects of microglia in MS are shown in Figure 3.

## 8. Negative Effects of Microglia in MS

As mentioned above, aside from the beneficial properties of microglia in the pathophysiology of MS, adverse effects are also documented. It is reasonable to distinguish several main mechanisms by which microglia exert their destructive effects in MS. First, microglia secrete many compounds, including cytokines, which have been shown to have neurotoxic effects in experimental tissue culture studies [64,71,72,73,74,75,76]. Proteases with such effects include cathepsin C, calpains, kallikrein 6, myeloperoxidase and matrix metalloproteinases [71,76]. Microglia are also responsible for secreting other compounds with cytotoxic effects, which include pro-inflammatory cytokines such as IFN-γ, TNF-α, IL-1β, IL-6, IL-12, semaphorin 4A, glutamate and reactive oxygen species [5,17,56,57,72,73,74,75,77,78,79,80]. In experimental studies, under the influence of these compounds, microglia induced the destruction of oligodendrocyte progenitor cells through TNF-α-induced death of oligodendrocytes, while the influence of IFN-γ destroyed neurons [64,73,74,77]. Of note, in the progressive form of MS, increased TNF-α expression is associated with increased expression of TNF receptor 1 (TNFR1), which promotes apoptosis and necrosis. However, it is not related to increased expression of TNFR2, which promotes cell viability [64,81]. 

As regards oxidative stress, microglia are responsible for the negative effects via mitochondrial injury, elevation of NADPH oxidase and nitric oxide synthase and downregulation of antioxidant enzymes [64,77,78,79]. The adverse effects are also mediated by microglia via ferrous iron recycling, which contributes to increased oxidative stress and CNS damage [64,82,83]. As a result, these CNS mitochondrial damages are involved in the pathogenesis of neurodegenerative diseases [84,85]. Changes in the state of microglia are involved in mitochondrial dysfunction and impaired regeneration in the CNS [86].

Another mechanism of the negative effects of microglia in MS is the change of specific cell types, which results in damaging effects. These processes can occur via secreted cytokines [64,87]. It has been shown that the generation of neurotoxic C3-positive astrocytes occurs through the release of TNF-α and IL-1β by microglia [64,87]. These astrocytes are responsible for enhancing the destruction of oligodendrocytes and neurocytes and are present in MS lesions [64,87]. Microglia also interact with T cells, leading to their activation, resulting in detrimental effects [64,88,89,90]. Negative effects can also be exerted by the secretion of certain chemokines by microglia. These include growth factors, which then mediate adverse events [64,91]. The negative effects of microglia in MS are presented in Figure 2.

## 9. Microglia and Remyelination

In MS and other demyelinating diseases, there is an imbalance between demyelination and remyelination, which can result in neurodegeneration [92]. Currently, the treatments for MS target the inflammatory process of the CNS. However, there are no therapies that can remyelinate and stop the progression of the disease [93,94]. OPCs are multipotent cells widely present in the CNS, which can differentiate into mature oligodendrocytes, resulting in remyelination [93]. In MS, the differentiation of OPCs into mature oligodendrocytes is impaired, which results in demyelination, deposition of myelin debris and the occurrence of axonal injury [93]. The clinical equivalent of this process is disability [93,95]. Therefore, the effective removal of remnant myelin resulting from demyelination is necessary for OPCs to differentiate into mature oligodendrocytes. It is also necessary for remyelination [93]. Microglia have a crucial role in the phagocytosis of these remnants and therefore also play a key role in remyelination [93]. Aside from the phagocytosis of myelin remnants, microglia also promote remyelination by secreting regenerative factors that are involved in promoting the same process [17,93]. An essential modulator affecting microglial functionality is the triggering receptor expressed on myeloid cells-2 (TREM2) [93]. This receptor responds to phospholipids. It is responsible for the activation of microglia as a result of demyelination and amyloid plaques in Alzheimer’s disease [93,96]. This entails an opportunity for new therapeutic possibilities. Studies have shown that the use of novel TREM2 agonist antibodies in the cuprizone model of CNS demyelination resulted in accelerated removal of myelin debris by microglia. Furthermore, there was an increase in OPC density in areas of demyelination [93]. This, in turn, led to an increase in the differentiation of mature oligodendrocytes, which enhanced demyelination and improved axonal integrity [93]. It demonstrates that TREM2 in microglia seems to be an interesting target for enhancing remyelination.

However, inadequate activation and recruitment of microglial cells as a result of demyelination may lead to the production of toxic mediators by microglia [92]. This causes the disruption of the remyelination process and results in increased demyelination [92]. Hence, it highlights the dualistic role of microglia, also in the remyelination process.

## 10. Impact of Disease-Modifying Therapies on Microglia

Disease-modifying therapies (DMTs) are a key aspect in the treatment of MS. DMTs slow disease progression and reduce the number of relapses in the relapsing forms of MS [97]. The current treatments mainly target inflammation [97]. Examples of widely known DMTs include dimethyl fumarate, glatiramer acetate, fingolimod, teriflunomide, ocrelizumab and interferons [97]. Undoubtedly, the currently used drugs are effective. However, since DMTs mainly target inflammation, more new drugs are sought to target another aspect of MS pathophysiology [93,94].

The significant role of microglia in the pathophysiology of MS is clear. Therefore, the search for new therapeutic options affecting microglia seems crucial. Currently, there are several studies with new drugs, i.e., Bruton’s tyrosine kinase inhibitors (BTKIs), which affect B cell activation and function [98,99,100,101]. B cells undoubtedly play a central role in the pathogenesis of MS, as has been seen with the successive implementation of monoclonal antibodies responsible for the depletion of B lymphocytes, such as rituximab [102]. BTKI is a key molecule involved in intracellular signaling from the B cell receptor and receptors of cells that are part of the innate immune system [102]. Thus, it can be suggested that BTKI may be more successful because it does not deplete B cells and affects other immune cells involved in the pathophysiology of MS [102]. BTKI is among the enzymes that catalyze the phosphorylation reaction of tyrosine residues by utilizing ATP [102]. The resulting phosphorylated proteins have many cellular functions, participating in the activation and deactivation of other proteins involved in multiple biochemical cascades [102]. Aside from its effects on B cells, BTKI also affects the function of other cells involved in the pathogenesis of MS, including dendritic cells, monocytes, macrophages and, most importantly, microglia [102]. Of note, by penetrating the brain, BTKI can exert effects on the inflammation and neurodegeneration of the CNS, which occurs by affecting B cells and microglia [98]. It was found that most CNS cells expressing BTKIs were microglia [101]. Experimental studies in vitro and in animals have shown that BTKIs downregulate pro-inflammatory cytokines of microglia [99,100]. Ibrutinib, a BTKI, inhibits lipopolysaccharide-induced M1 activation in BV2 microglial cells and wild-type mice, resulting in the inhibition of neuroinflammation mediated by microglia [99]. Moreover, experimental studies have shown that BTKIs can favor remyelination, which is achieved by influencing microglia [101]. This indicates the availability of a novel and highly promising therapeutic possibility. However, the exact cellular mechanism of how BTKIs promote remyelination has yet to be determined [101]. 

In a randomized phase 2b study, Reich et al. investigated the use of tolebrutinib in patients with the relapsing form of MS [103]. Tolebrutinib is an oral reversible brain-penetrating BTKI [103]. Twelve weeks of therapy with this drug resulted in a dose-dependent reduction in new gadolinium-enhancing lesions [103]. Given the reduction in acute inflammation and the potential ability to modulate the immune response in the CNS, this is a promising study, and it seems reasonable to investigate the effects of this drug on microglia [103]. 

As mentioned earlier, BTKIs affect the function of myeloid cells. The phagocytic capacity of macrophages is mediated by FcγR, while the secretion of the cytokines IL1b and TNF-α is mediated by TLR4 [102]. BTKIs are involved in the signaling that occurs after the stimulation of these receptors [102,104]. Importantly, using ibrutinib, it was shown that BTKI activity led to the inhibition of FcγR-mediated TNF-α release. However, phagocytic capacity was not affected [102,105]. Studies on monocytes from healthy volunteers are also promising since, they showed that evobrutinib, which is an irreversible BTKI, was responsible for polarizing macrophages toward the M2 phenotype with beneficial (i.e., anti-inflammatory) properties [102]. In mouse models of Alzheimer’s disease, BTKIs were responsible for modulating phagocytosis by microglia [106]. This also suggests that a similar phenomenon may occur in MS. 

It is important to emphasize the need to seek other therapies as well. It is known that neurodegeneration in MS involves the gray matter [5,61,62]. This is an irreversible process and is mainly responsible for permanent disability in MS [5,61,62]. Currently, the available treatments for MS affect the white matter, whereas they are of limited use in terms of the loss of the gray matter [5,61,62,63]. This suggests the necessity of searching for new therapies that could stop this process. Of note, microglia participate in the pathological process of the gray matter in MS [5]. Therefore, it is important to conduct further research on microglia and the cytokines they secrete, which would potentially provide insight into new therapeutic options for MS. It could allow a breakthrough in the management of this condition.

## 11. Potential Use of Microglia Cytokines as Markers of MS

It is important to note that microglia cytokines might potentially prove to be useful clinical markers. Currently, however, there is a gap in the literature on this issue. Nevertheless, it is necessary to know the relevant correlations between the concentrations of these cytokines, e.g., in plasma, and clinical parameters, such as the expanded disability status scale (EDSS), or radiological parameters, such as the number of T2 hyperintense lesions in magnetic resonance images of the head [107]. It might then be possible in clinical practice to determine the concentrations of individual cytokines, reflecting the activity of the disease process. Clearly, it is necessary to research in this regard.

## 12. Conclusions

Understanding more precisely the role of microglia-associated cytokines in MS pathophysiology is currently a novel and important research topic. Microglia are involved in a variety of significant effects in MS, such as the release of many protective and negative substances. The protective effects include the secretion of growth factors with regenerative effects promoting cell survival, proliferation and maturation. The elimination of destructive T cells is another beneficial effect. However, microglia also exert many negative effects, such as the secretion of cytotoxic compounds, the exacerbation of oxidative stress and the promotion of neurotoxic astrocytes and T cells. Microglia mediate many of these processes via the secretion of cytokines. 

Despite the important role of microglia in MS pathophysiology, there is currently a gap in the literature regarding the relationship between microglial cytokines and clinical and radiological parameters. Irreversible neurodegeneration of the gray matter occurs in the early stages of MS and is mainly responsible for the disability of patients. Current disease-modifying treatments have only a limited effect on this process. Therefore, determining the relationship between the plasma concentrations of microglia-associated cytokines and the clinical status and radiological findings may prove crucial not only for a better understanding of MS pathophysiology but also for a potential indication of the target points for a breakthrough in MS therapies that could perhaps limit the loss of the white matter and neurodegeneration of the gray matter, which is of crucial importance to disability in MS. It is also important to search for MS drugs that can modulate microglia functions (anti-inflammatory and pro-regenerative effects). Further studies are warranted on the relationship between microglia and BTKIs, which are a promising group of MS drugs.

## Figures and Tables

**Table 1:** Comparison of different microglial phenotypes.

	M1 Microglia	M2 Microglia			M3 Microglia
		M2a	M2b	M2c	
Mediators responsible for activation	lipopolysaccharide IFN-γ	IL-4 IL-13	TLR and FCγ receptor fusion	IL-10 TGF-β glucocorticoids	The mediators responsible for activating M1 or M2, depending on the situation
Function	Neurotoxicity Release of pro-inflammatory agents Induction of inflammation	Anti-inflammatory effect Antiparasitic effect Phagocytosis Collagen formation Tissue repair	Immunoregulation M1-like effect Recruitment of regulatory T cells Release of IL-10	Immunosuppressive effect Immunoregulation Matrix remodeling Tissue repair	Shift in M1 or M2, depending on the inducing agents

## Data Availability

Not apllicable.
